# Microbiome‐mediated alleviation of tobacco replant problem via autotoxin degradation after long‐term continuous cropping

**DOI:** 10.1002/imt2.189

**Published:** 2024-04-02

**Authors:** Peixue Xuan, Haikun Ma, Xiaopeng Deng, Yunfu Li, Jianqing Tian, Junying Li, Erdeng Ma, Zhaoli Xu, Dong Xiao, T. Martijn Bezemer, Mingfeng Wang, Xingzhong Liu, Meichun Xiang

**Affiliations:** ^1^ State Key Laboratory of Mycology, Institute of Microbiology Chinese Academy of Sciences Beijing China; ^2^ University of Chinese Academy of Sciences Beijing China; ^3^ Department of Microbiology, College of Life Science Nankai University Tianjin China; ^4^ Yunnan Academy of Tobacco Agriculture Science Kunming China; ^5^ Institute of Botany Chinese Academy of Sciences Beijing China; ^6^ Research and Development Center China Tobacco Yunnan Industrial Co., Ltd. Kunming China; ^7^ Aboveground‐Belowground Interactions Group, Institute of Biology Leiden University Leiden The Netherlands

## Abstract

Continuous cropping often results in severe “replant problem,” across various crops due to the autotoxins accumulation, soil acidification, pathogens proliferation, and microbial dysfunction. We unveiled a groundbreaking phenomenon that long‐term continuous cropping (LTCC) can alleviate the tobacco replant problem. This mitigation occurs through the enrichment of autotoxin‐degrading microbes, and the transformative impact is evident with even a modest application (10%) of LTCC soil to short‐term continuous cropping (STCC) soil. Our investigation has pinpointed specific autotoxin‐degrading bacteria, particularly the *Pseudomonas* and *Burkholderia* species, which exhibit the capacity to alleviate the tobacco replant problem in STCC soil. Their autotoxin‐degrading mechanism using axenic culture and soil samples was also conducted via comprehensive analyses of microbiome and transcriptome approach. This research sheds light on the potential of LTCC as a strategic approach for sustainable agriculture, addressing replant problems and promoting the health of cropping systems. UV, ultraviolet; OD, optical density.

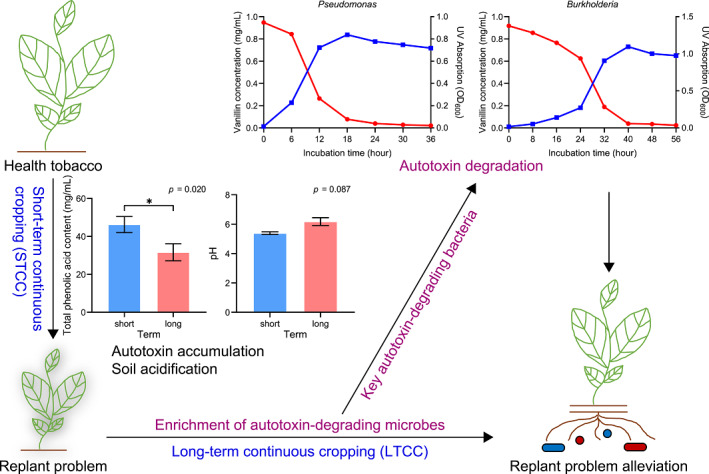

Many crops suffer from replant problems that often are caused by biotic and abiotic factors in the soil, such as autotoxicity, disorder in physicochemical soil properties (such as changes in pH), or the build‐up of pathogens in the soil [1, 2]. One of the best‐explored causes of replant problems in many crops is autotoxicity. Plants release toxic chemicals into the environment via root exudates or degrading plant residues [3]. Many types of phenolic acids found in plants and in the environment surrounding the plant can have phytotoxic effects, leading to replant problems and yield losses [4]. Vanillin is an autotoxin affecting several plant species, which can significantly increase the abundance of pathogenic fungi in soil [5].

Although many studies have documented the negative effects of continuous cropping, several studies have shown that repeated planting of the same crop can lead to beneficial adaptations, such as the development of disease‐suppressive soil [6]. Long‐term monocultures of certain crops can regulate the composition and function of the rhizosphere microbiome, promoting its ability to suppress fungal pathogens [6]. This modulation of soil microbial communities by plants as an adaptation mechanism has been described as a “cry for help” strategy, by which plants can recruit beneficial microbes to enhance their ability to relieve stresses and cope with environmental changes [7].

Tobacco (*Nicotiana tabacum* L.) is an economic crop that is planted all over the world. Yunnan is the largest tobacco production region in China, and tobacco is replanted every year with intercropping of other crops. This continuous cropping regime of tobacco has resulted in a serious replant problem which results in poor growth and development of plants due to soil acidification and the accumulation of autotoxins in the soil (mainly phenolic acids), and this has been linked to microbial community dysfunction [3]. However, there are no published reports on the alleviation of this replant problem after long‐term continuous cropping (LTCC). During a survey mapping the tobacco replant problem in Yunnan, local scientists and farmers in the tobacco area observed that, while the replant problem is a substantial issue in soil used for short‐term continuous cropping (STCC; ≤5 years), soil used for LTCC (≥10 years) display no serious replant problems (Figures S1 and S2). Given that replant problems in tobacco have been linked to autotoxin accumulation in the soil [3], we hypothesize that LTCC may result in adaptation within the microbiota, including the accumulation of microbial populations capable of breaking down autotoxins in the soil, thereby alleviating replant problems. Here, we report that serious replant problems in tobacco STCC soil are associated with higher amounts of phenolic acids, especially vanillic acid, vanillin, and cinnamic acid in the soil (Table S1). We show that vanillin in particular is highly hazardous to tobacco seeds, even at low concentrations, and that the growth of tobacco seedlings is significantly affected in soil containing vanillin (Figures S3 and S4).

## RESULTS AND DISCUSSION

**Figure 1:**
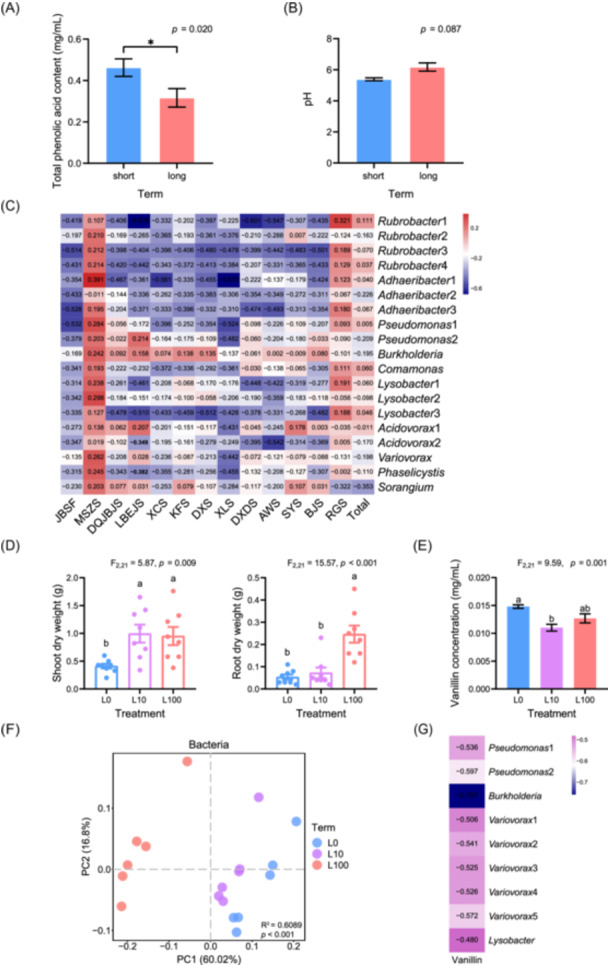
Long‐term continuous cropping (LTCC) of tobacco modulates soil acidification, autotoxin accumulation, and root‐associated microbiota. (A) Total phenolic acids content in tobacco field soil. (B) Soil pH value in tobacco field soil. Means are compared using a linear mixed model. Error bars represent standard errors (short‐term continuous cropping, STCC, *n* = 18; LTCC, *n* = 16), * indicates *p* < 0.05. (C) Heatmap of Spearman's correlation coefficients correlating bacterial taxa differentially abundant in LTCC soil and phenolic acid. (D) Shoot and root dry weight of tobacco seedlings in STCC (L0), a mixture of 10% LTCC and 90% STCC (L10) and LTCC (L100) soil (*n* = 8). (E) Phenolic acid (vanillin) content in L0, L10, and L100 soil (*n* = 8). (F) Principal coordinates analysis (PCoA) of bacterial community structure (metagenomic sequencing) in L0, L10, and L100 soil (*n* = 6). (G) Heatmap of Spearman's correlation coefficients between vanillin and the bacterial taxa (metagenomic sequencing) differentially abundant in L100 and L10 soil treated with autotoxin. AWS, ferulic acid; BJS, benzoic acid; DQJBJS, *p*‐hydroxybenzoic acid; DXDS, *p*‐coumaric acid; DXS, syringic acid; KFS, caffeic acid; JBSF, phloroglucinol; LBEJS, phthalic acid; MSZS, gallic acid; RGS, cinnamic acid; SYS, salicylic acid; Total, total phenolic acids; XCS, vanillic acid; XLS, vanillin.

External challenges can stimulate plants to employ a “cry for help” strategy, a mechanism by which plants recruit beneficial microbes to enhance their ability to relieve stress and facilitate their adaptation to environmental changes [8]. A comparison between field soil of LTCC (16 fields) and STCC (18 fields) shows that LTCC is associated with a reduction in autotoxins content (such as phenolic acids) and soil acidification (Figure 1A,B), resulting in an alleviation of some of the negative impacts on soil associated with continuous cropping. These alterations are also associated with changes in the microbial community in soil (Figures S5 and S6). Operational taxonomic units (OTUs) belonging to the genera *Pseudomonas, Burkholderia, Rubrobacter, Adhaeribacter, Comamonas, Lysobacter, Acidovorax, Variovorax, Phaselicystis, Sorangium, Trichoderma, Preussia, Arcuadendron*, and *Penicillium* were significantly more abundant in LTCC soil (Table S2), and the relative abundance of *Pseudomonas* (OTU_831 and OTU_2553), *Rubrobacter* (OTU_3186, OTU_4470, OTU_3533, and OTU_9018), *Adhaeribacter* (OTU_6752, OTU_6780, and OTU_6878), *Comamonas* (OTU_1967), *Lysobacter* (OTU_1591, OTU_1786, and OTU_2415), *Acidovorax* (OTU_1226 and OTU_2942), *Variovorax* (OTU_2739), *Phaselicystis* (OTU_4233), *Sorangium* (OTU_3079), and *Trichoderma* (OTU_1474), *Preussia* (OTU_2198), *Arcuadendron* (OTU_1567), and *Penicillium* (OTU_2457 and OTU_249) were significantly negatively correlated with the concentration of phenolic acids in soil (Figures 1C and S7). These results suggest that tobacco might employ a “cry for help” strategy in which high amounts of phenolic acids stimulate tobacco plants to promote the growth of beneficial soil microbes that are capable of degrading phenolic acids. Our finding indicates that LTCC soil resembles the patterns observed for other crop monocultures with suppressive soil [6]. Exposure to long‐term stresses can induce soil homeostasis against biotic and abiotic stresses in both LTCC and monocultures. Our study shows that LTCC‐associated microbiota can help alleviate the tobacco replant problem associated with STCC soil.

Microbiota‐mediated disease suppression can be transferred from suppressive soil to conducive soil [6]. Similarly, in a controlled experiment, we show that adding 10% LTCC soil to STCC soil (L10) reduces autotoxin content in the soil and promotes plant growth (Figures 1D,E and S8). This effect is associated with changes in autotoxin levels and in bacterial community composition (Figure 1F). The relative abundances of OTUs assigned to the genera *Pseudomonas, Burkholderia, Rubrobacter, Adhaeribacter, Comamonas, Lysobacter, Acidovorax*, and *Variovorax* were all significantly higher in LTCC soil (Table S3), which is in accordance with our previous results obtained with field soil (Table S2). Notably, the abundances of *Pseudomonas* (OTU_17288), *Lysobacter* (OTU_17914), and *Variovorax* (OTU_14122, OTU_14136, OTU_14118, OTU_14131, and OTU_14137) were significantly negatively correlated with the amount of vanillin in LTCC soil (Figure 1G), suggesting that some of these microbes may be capable of vanillin degradation. A similar pattern was found in L10 soil, where the genera *Pseudomonas, Burkholderia, Comamonas, Lysobacter, Acidovorax*, and *Variovorax* were significantly differentially abundant (Table S3), and where *Pseudomonas* (OTU_17295), *Burkholderia* (OTU_13611), and *Lysobacter* (OTU_17914) were significantly negatively correlated with vanillin levels (Figure 1G). This suggests that microbes capable of autotoxin degradation in LTCC soil were transferred to STCC soil, where they can relieve the replant problem. Such microbes with the ability to degrade autotoxins have previously been shown to exhibit beneficial effects on plant growth and soil health, as illustrated by *Pseudomonas, Burkholderia, Comamonas, Trichoderma, Acidovorax, Variovorax, Rubrobacter, Lysobacter, Adhaeribacter*, and *Preussia* species that can degrade phenolic acids, biodegradation potential or promote plant growth [9, 10].

**Figure 2:**
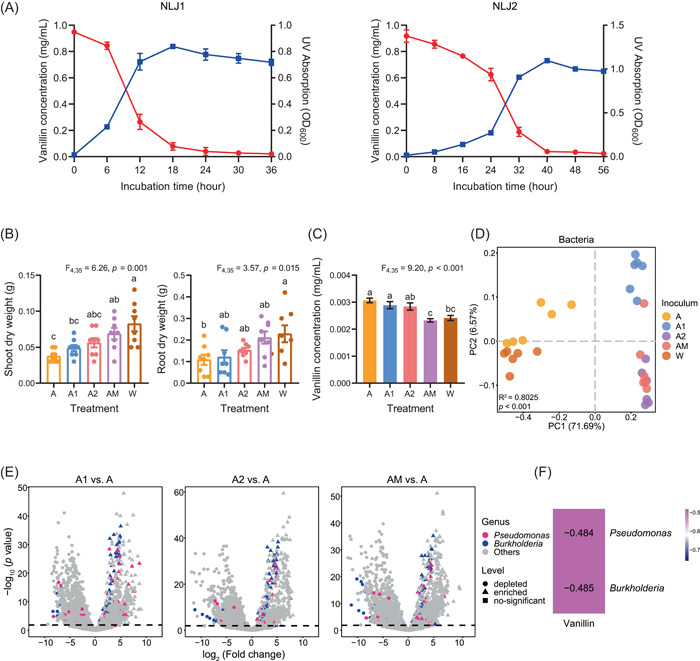
Autotoxin‐degrading bacteria alleviate the replant problem via the colonization and autotoxin degradation in soil. (A) Growth curves of two autotoxin‐degrading bacteria and dynamics of vanillin concentration along with the growth of the bacteria NLJ1 and NLJ2. Error bars represent standard errors (*n* = 4). (B) Shoot and root dry weight in the soil of water (W), autotoxin (A), autotoxin + NLJ1 (A1), autotoxin + NLJ2 (A2), autotoxin + NLJ1 + NLJ2 (AM) (*n* = 8). (C) Vanillin concentration in the soil treated with W, A, A1, A2, and AM (*n* = 8). (D) Principal coordinates analysis (PCoA) of bacterial community structure of the treatments of W, A, A1, A2, and AM (*n* = 6). (E) Volcano plot illustrating differentially abundant bacterial taxa of autotoxin‐degrading microbes in vanillin‐modified soil. Points are colored according to the classification of operational taxonomic units (OTUs) at the genus level. Point shapes represent OTUs that are enriched, depleted, or not significant in two groups. The dashed line indicates the −log_10_ (*p* value) which divides significantly and non‐significantly differentially abundant OTUs. (F) Heatmap of Spearman's correlation coefficients between vanillin and the bacterial taxa differentially abundant in short‐term continuous cropping soil treated with autotoxin‐degrading microbes. OD, optical density; UV, ultraviolet.

Analysis of the microbiome data revealed that *Pseudomonas* and *Burkholderia* species were significantly more abundant in both native LTCC soil and STCC soil amended with 10% LTCC soil (Table S3) and that their abundances were significantly negatively correlated with the amounts of phenolic acids in the soil (Figure 1G). We further isolated two species (i.e., NLJ1 and NLJ2) from LTCC soil using a mineral salt medium (MSM) with vanillin as the sole carbon. Phylogenetic tree analysis revealed that NLJ1 is most closely related to *Pseudomonas plecoglossicida* (NR114226), and NLJ2 to *Burkholderia pyrrocinia* (NR029210) (Figure S9). Both isolates were able to efficiently degrade vanillin in axenic culture and in STCC soil (Figure 2A,C). Furthermore, inoculation of NLJ1, NLJ2, or their combination into vanillin‐modified STCC soil where the growth of tobacco seedlings was significantly inhibited, alleviates the tobacco replant problem (Figures 2B and S4). The bacterial community in the soil changed after the inoculation of either or both strains into the vanillin‐modified STCC soil (Figure 2D). The abundances of the two autotoxin‐degrading bacteria significantly increased, indicating that these two bacteria can colonize soil well and establish populations in the soil (Figures 2E and S10 and Tables S4 and S5). Metagenome analysis also showed that *Pseudomonas* (OTU_14268) and *Burkholderia* (OTU_10908) significantly negatively correlated with vanillin levels in the soil after inoculation (Figure 2F).

The degradation of vanillin by bacteria has not been well illustrated in the literature. Vanillin can be indirectly degraded through the degradation of phenolic acids or aromatic compounds, while only a limited number of strains are known to directly degrade autotoxin [11]. In this study, we observed significant upregulation of genes associated with autotoxin degradation in the autotoxin‐degrading bacteria *Pseudomonas* and *Burkholderia* under vanillin stress in axenic cultures of MSM (Table S6). However, we acknowledge that vanillin degradation in soil is complex, and we recognize that its degradation may involve more intricate mechanisms. The upregulation of glyoxylate and dicarboxylate metabolism plays a crucial role in the degradation of aromatic compounds [12]. In our study, we observed upregulation of glyoxylate and dicarboxylate metabolism in both axenic cultures and in LTCC soil after vanillin addition (Table S6). This pathway might be involved in vanillin degradation or, at the very least, in the bacterial response to vanillin‐induced stress. Our study uncovers upregulated expression of the functional genes involved in the colonization of and vanillin degradation by *Pseudomonas* and *Burkholderia* and illustrates that LTCC alleviates the STCC replant problem of tobacco via enriching antistress and autotoxin‐degrading microbiota.

## CONCLUSION

In summary, soil acidification and phenolic acid accumulation have been considered the main causes of replant problems for many crops. We demonstrate that the LTCC of tobacco alleviates replant problems by increasing soil pH and decreasing phenolic acids in the soil via enrichment of autotoxin‐degrading bacteria. The potent effects of tobacco autotoxin‐degrading bacteria are further confirmed through validations in both axenic cultures and soil samples. These findings highlight that LTCC or monocultures of crops under certain stresses can induce soil homeostasis against replant problems, soil‐borne diseases, and potentially other stresses to increase crop health.

## AUTHOR CONTRIBUTIONS

Meichun Xiang, Xingzhong Liu, Peixue Xuan, Haikun Ma, and Xiaopeng Deng conceived and designed the study. Peixue Xuan, Haikun Ma, Xiaopeng Deng, Yunfu Li, Jianqing Tian, Junying Li, Erdeng Ma, Zhaoli Xu, and Dong Xiao collected the samples and conducted the preliminary analysis. Peixue Xuan performed the experiments, analyzed the data, and wrote the manuscript. Haikun Ma, T. Martijn Bezemer, Mingfeng Wang, Xingzhong Liu, and Meichun Xiang revised the manuscript. All authors have read the final manuscript and approved it for publication.

## CONFLICT OF INTEREST STATEMENT

The authors declare no conflict of interest.

## ETHICS STATEMENT

No animals or humans were involved in this study.

## Supporting information


**Figure S1**: Tobacco plant growth in field soil in long‐term continuous cropping (LTCC) and short‐term continuous cropping (STCC) (n = 3).
**Figure S2**: Location, distribution and tobacco growth of sampling sites in field soil.
**Figure S3**: Effects of vanillin and degrading microbes on tobacco seeds germination and growth (n = 4).
**Figure S4**: Vanillin degradation and tobacco seedling growth in short‐term continuous cropping (STCC) soil by inoculation of autotoxin‐degrading bacteria NLJ1, NLJ2 and their combination (n = 8).
**Figure S5**: Microbial community structure in tobacco field soil in long‐term continuous cropping (LTCC, n = 16) and short‐term continuous cropping (STCC, n = 18).
**Figure S6**: Microbial community composition in tobacco field soil in long‐term continuous cropping (LTCC, n = 16) and short‐term continuous cropping (STCC, n = 18).
**Figure S7**: Heatmap of Spearman's correlation coefficients correlating fungal taxa enriched in long‐term continuous cropping soil and phenolic acid.
**Figure S8**: Effects of autotoxin on tobacco seedling growth in soil treated with autotoxin in different continuous cropping years (n = 8).
**Figure S9**: Phylogenetic tree of two strains and their closest sequences in GenBank based on 16S rRNA gene sequences.
**Figure S10**: Sequence alignment using 16S rRNA gene sequence of strain NLJ1 against gene sequence from metagenome sequencing in soil by inoculation of autotoxin‐degrading bacteria.

**Table S1**: Comparison of phenolic acid contents in tobacco field soil in different continuous cropping years.
**Table S2**: Bacterial and fungal taxa differentially abundant in long‐term continuous cropping (LTCC) soil.
**Table S3**: Bacterial taxa differentially abundant in L100 (long‐term continuous cropping) and L10 (short‐term continuous cropping added with 10% (w/w) of long‐term continuous cropping) soil treated with autotoxin.
**Table S4**: Bins in metagenome‐assembled genomes differentially abundant in short‐term continuous cropping (STCC) soil treated with mixed strains.
**Table S5**: Gene differentially abundant in short‐term continuous cropping (STCC) soil treated with mixed strains.
**Table S6**: Functional categories of differentially enriched genes related to vanillin degradation in both axenic culture and long‐term continuous cropping (LTCC) soil with vanillin addition.
**Table S7**: Locations and distributions of sampling sites in tobacco field soil.
**Table S8**: Standard curve equations of thirteen phenolic acids.

## Data Availability

DNA sequences of isolates are submitted to GenBank with the following accession numbers OM968489 (https://www.ncbi.nlm.nih.gov/nuccore/OM968489) and OM969808 (https://www.ncbi.nlm.nih.gov/nuccore/OM969808). The amplicon sequence data have been submitted to Sequence Read Archive (SRA) of National Center for Biotechnology Information database under the BioProject ID PRJNA814864 (https://www.ncbi.nlm.nih.gov/bioproject/PRJNA814864) and PRJNA814872 (https://www.ncbi.nlm.nih.gov/bioproject/PRJNA814872). Transcriptome sequencing data are available at SRA under the BioProject number PRJNA928220 (https://www.ncbi.nlm.nih.gov/bioproject/PRJNA928220). Metagenome sequencing data can be freely accessed at SRA under the BioProject number PRJNA935121 (https://www.ncbi.nlm.nih.gov/bioproject/PRJNA935121). The data and scripts used are saved in GitHub (https://github.com/PeixueXuan/Microbial-alleviation-of-replant-problem.git). Supplementary materials (methods, figures, tables, scripts, graphical abstract, slides, videos, Chinese translated version, and updated materials) may be found in the online DOI or iMeta Science http://www.imeta.science/.
