# Supplementary material for: Microbiome‐mediated alleviation of tobacco replant problem via autotoxin degradation after long‐term continuous cropping
Source: Imeta. 2024 Apr 2;3(2):e189. doi: 10.1002/imt2.189 (PMC11170962; doi:10.1002/imt2.189)
Supplement: Supplementary file 1 — Figure S1: Tobacco plant growth in field soil in long‐term continuous cropping (LTCC) and short‐term continuous cropping (STCC) (n = 3). Figure S2: Location, distribution and tobacco growth of sampling sites in field soil. Figure S3: Effects of vanillin and degrading microbes on tobacco seeds germination and growth (n = 4). Figure S4: Vanillin degradation and tobacco seedling growth in short‐term continuous cropping (STCC) soil by inoculation of autotoxin‐degrading bacteria NLJ1, NLJ2 and their combination (n = 8). Figure S5: Microbial community structure in tobacco field soil in long‐term continuous cropping (LTCC, n = 16) and short‐term continuous cropping (STCC, n = 18). Figure S6: Microbial community composition in tobacco field soil in long‐term continuous cropping (LTCC, n = 16) and short‐term continuous cropping (STCC, n = 18). Figure S7: Heatmap of Spearman's correlation coefficients correlating fungal taxa enriched in long‐term continuous cropping soil and phenolic acid. Figure S8: Effects of autotoxin on tobacco seedling growth in soil treated with autotoxin in different continuous cropping years (n = 8). Figure S9: Phylogenetic tree of two strains and their closest sequences in GenBank based on 16S rRNA gene sequences. Figure S10: Sequence alignment using 16S rRNA gene sequence of strain NLJ1 against gene sequence from metagenome sequencing in soil by inoculation of autotoxin‐degrading bacteria. [file IMT2-3-e189-s002.docx]

**Supporting information to:**

**Microbiome-mediated alleviation of tobacco replant problem via autotoxin degradation after long-term continuous cropping**

**Running title**: Microbial alleviation of replant problem

Peixue Xuan^1,2#^, Haikun Ma^3#^, Xiaopeng Deng^4#^, Yunfu Li^1,2^, Jianqing Tian^5^, Junying Li^4^, Erdeng Ma^4^, Zhaoli Xu^4^, Dong Xiao^6^, T. Martijn Bezemer^7^, Mingfeng Wang^6^*, Xingzhong Liu^1,3^*, Meichun Xiang^1,2^*

^1^State Key Laboratory of Mycology, Institute of Microbiology, Chinese Academy of Sciences, Beijing 100101, China

^2^University of Chinese Academy of Sciences, Beijing 100049, China

^3^Department of Microbiology, College of Life Science, Nankai University, Tianjin 300071, China

^4^Yunnan Academy of Tobacco Agriculture Science, Kunming 653100, China

^5^Institute of Botany, Chinese Academy of Sciences, Beijing 100093, China

^6^Research and Development Center, China Tobacco Yunnan Industrial Co., Ltd., Kunming 650231, China

^7^Institute of Biology, Aboveground-Belowground Interactions Group, Leiden University, Leiden 2333 BE, The Netherlands

^#^These authors contributed equally to this work.

*Corresponding authors.

Email address: wangmf@ynzy-tobacco.com (Mingfeng Wang); liuxz@nankai.edu.cn (Xingzhong Liu); xiangmc@im.ac.cn (Meichun Xiang)

**Methods**

**Experiment 1: Effects of continuous cropping on soil pH, autotoxins and root-associated microbial communities in tobacco field soil**

**Field soil sampling**

Three tobacco growing locations with different continuous cropping years in tobacco growing regions in Yunnan Province of China were selected for sampling. A total of 34 soil samples from 18 fields with short-term (3 or 5 years), and 16 with long-term (20 years) cropping were collected in July 2020 in the vigorous growth stage of tobacco (Figure S2 and Table S7). Each soil sample was a mixture of root-associated soil collected from five plants per field. The soil samples were taken with a shovel at 0-15 cm depth across an area of 5000 m^2^ in each tobacco field.

2 g soil from each sample was transferred into sterile cryopreservation tubes and immediately frozen in liquid nitrogen. These tubes were transported to the laboratory with dry ice and stored at -80°C for future DNA extraction. The remnant was kept in sterile plastic bags on ice. These soil samples were sieved through a 2.0-mm mesh and divided into two parts to analyze soil pH and phenolic acids. All samples were stored at 4°C before further analysis.

**Determination of soil pH and phenolic acids**

Soil pH was measured using a pb-10 pH meter (Sartorius, Germany). Phenolic acids were extracted from soil samples with the method previously described [1]. Fresh soil samples (25 g) were placed into a centrifuge tube, and 1 M NaOH was added. Subsequently, the tubes were stored for 24 h at 25°C in an incubator. The suspension was centrifuged to separate the liquid supernatant. The pH of the supernatant was acidified to 2.5 with 12 M HCl and stored for 2 h. The supernatant was stored at 4°C after centrifugation. The solution was filtrated through a 0.22-μm microporous membrane filter.

High-performance liquid chromatography (HPLC) analysis was used to identify soil phenolic acids [2]. The analytical conditions were as follows: instrument: Agilent 1260 (Agilent, USA), chromatographic column: XDB-C18 (4.6 mm × 250 mm), the temperature of the column: 40°C, velocity of flow: 1.0 mL/min, ultraviolet (UV) detection wavelength: 230 nm, injection volume: 50 μL. Methyl cyanide (A) and acetic acid solution (B) were used as mobile phases with a gradient elution B: 95% (0 min) → 85% (25 min) → 85% (30 min) → 65% (50 min) → 0% (50.5 min) → end (60 min). Phenolic acids were determined based on the retention times of each phenolic acid, and the concentrations of each phenolic acid were quantified based on peak areas of pure standards using external standards. The standard phenolic acids used for HPLC analysis were phloroglucinol, gallic acid, *p*-hydroxybenzoic acid, phthalic acid, vanillic acid, caffeic acid, syringic acid, vanillin, *p*-coumaric acid, ferulic acid, salicylic acid, benzoic acid and cinnamic acid (Shanghai yuanye Bio-Technology Co., Ltd, China). All the chemicals procured were of HPLC spectral grade. Phenolic acids contents were calculated based on standard curve equations (Table S8).

**Amplicon sequencing and bioinformatic analyses**

Total soil DNA was extracted from 0.25 g freeze-dried soil samples using DNeasy^®^ PowerSoil^®^ Kit (Qiagen Inc., Germany) following the manufacturer’s protocol. The purity and concentration of DNA have been checked on 1% agarose gels by electrophoresis and NanoDrop spectrophotometer (Thermo Fisher Scientific, USA).

The V3-V4 hypervariable region of the bacterial 16S ribosomal RNA (rRNA) gene was amplified with universal forward primer 338F and reverse primer 806R [3]. The fungal internal transcribed spacer (ITS) region was amplified with universal forward primer ITS1F and reverse primer ITS2 [4]. The PCR was carried out on Mastercycler Gradient (Eppendorf, Germany). The amplified PCR products were confirmed via electrophoresis on 1% agarose gels. The bands were purified using Agencourt^®^ AMPure^®^ XP Kit (Beckman, USA). The NEB Next^®^ Ultra™ DNA Library Prep Kit for Illumina from New England Biolabs Company (USA) was used to construct a DNA library. The amplicon library was sequenced by the Illumina^®^ MiSeq^®^ platform (Illumina Inc., USA).

The raw data were screened and sequences shorter than 230 bp and sequences with low-quality scores (≤ 20) were removed. Qualified reads were clustered into the same operational taxonomic units (OTUs) at a similarity level of 97% using UPARSE v7.1 [5] and the algorithm of VSEARCH v2.7.1. The SILVA database release 138 [6] was used to classify bacterial sequences into different taxonomic groups. The taxonomic assignment for fungi was based on the BLAST algorithm [7] through UNITE database [8]. OTU tables were generated to record the reads of each OTU in each sample and its taxonomic affiliation. All samples were rarefied in the “vegan” package of R v3.6.1 [9].

**Experiment 2: Effects of long-term continuous cropping (LTCC) on the microbial communities of short-term continuous cropping (STCC) soil**

**Experimental design**

A soil inoculation experiment was conducted in a greenhouse of the Institute of Microbiology, Chinese Academy of Sciences, from June 2022 to August 2022. Soil samples were taken from Niulanjiang Country (25º17’ N, 103º13’ E), Yunnan Province, China. The tobacco variety “K326” was planted continuously for 3 years (STCC) and 20 years (LTCC) in fields. The soil collection method was the same as the above-mentioned. The treatments were: i) L0: 100% live STCC soil, ii) L10: 90% live STCC soil amended with 10% (w/w) of live LTCC soil, iii) L100: 100% live LTCC soil. Each pot contained 500 g soil. In total, there were 3 treatments × 8 replicates = 24 pots. The tobacco variety used was “K326” and had been cultivated in a floating seedling system. One-month-old tobacco seedlings were transplanted, one per pot. All pots were placed in a completely random design in the greenhouse (average temperature of 25℃, relative humidity of 60-80%, photoperiod of 16 h light and 8 h dark). Previous work showed that phenolic acids are rapidly depleted when added to soil owing to the presence of microorganisms [10]. Therefore, autotoxin (vanillin) was added to the soil weekly to maintain the desired level in this experiment. We found that vanillin concentration ranged from 0.0179 to 0.0223 mg/g dry soil in tobacco fields (Table S1). We added 0.05 mg/g dry soil of vanillin in soil to ensure the manifestation of significant effects on tobacco seedling growth and microbes under controlled conditions. The plants were watered every 2 d. The pot experiment was harvested 4 weeks after transplanting. Tobacco seedlings were destructively sampled and separated into shoots and roots. The plant height, leaf number, fresh weight and dry weight were measured. Root-associated soil was collected and handled simultaneously for autotoxin determination and microbial analysis as mentioned previously.

**Metagenome sequencing and bioinformatic analyses**

For metagenome sequencing, 6 replicate pots were chosen randomly from each treatment, with each pot contributing a unique sample. Total soil DNA was extracted from pot experiment samples using the DNeasy® PowerSoil® Kit. The purity and quality of the DNA were checked on 1% agarose gels. DNA concentrations were quantified using a Qubit 2.0 fluorometer. DNA was sheared to 300 bp using an ultrasonic crusher (Covaris, USA). To prepare the sequencing library, the fragments were treated by end repair, “A” tailing, and ligation of Illumina-compatible adapters. DNA sequencing libraries were deep sequenced on the Illumina NovaSeq platform.

The quality control of raw data was carried out using Trimmomatic [11], including the removal of adapter sequences and low-quality reads. High-quality sequences were assembled into contigs by using MEGAHIT [12]. Contigs with a length of at least 800 bp were selected as the final assembling results. A non-redundant gene catalog was constructed using CD-HIT [13]. Reads after quality control were mapped to a non-redundant gene catalog by using Bowtie 2 [14], and the abundance information of genes in each sample was counted. Representative sequences of the non-redundant gene catalog were annotated based on the non-redundant (NR) protein database [15] using DIAMOND [16]. Gene function was annotated by searching against the Kyoto Encyclopedia of Genes and Genomes (KEGG) [17].

Binning analysis was performed using Vamb [18], and the results of bins were evaluated using CheckM [19] to extract ID, taxonomy, completeness, and containment information. The bins results were screened, and the screening criteria were completeness > 50% and contamination < 10%.

**Experiment 3: Determination of the ability of autotoxin-degrading microbes differentially abundant in LTCC soil to degrade autotoxins**

**Isolation and identification of LTCC-differentially abundant microbes**

Isolation and purification of microbes from LTCC tobacco soil were carried out using the method previously described [20]. Vanillin at 1 mg/mL was added to mineral salt medium (MSM) as the sole carbon source. Identification of purified bacteria was based on 16S rRNA gene sequences with universal forward primer 27F and reverse primer 1492R. The amplified PCR products were verified by electrophoresis on 1% agarose gels, and then sequenced. Phylogenetic and molecular evolutionary analyses of strains were conducted using MEGA version X [21]. Other differentially abundant taxa were found in LTCC soil but we failed to establish cultures for these taxa. Therefore, we finally chose two isolates (NLJ1 and NLJ2) for subsequent experiments.

**Phenolic acid degradation assay of LTCC-differentially abundant microbes**

Tests of microbial growth and phenolic acid degradation were carried out using the method previously described [20]. MSM containing different concentration of vanillin was prepared (0.01, 0.02, 0.03, 0.04, 0.05, 0.06 mg/mL) for the calculation of standard curve equations. The OD_600_ and OD_250_ values (optical density, OD) were detected for the samples taken from cultures using a SmartSpec 3000 spectrophotometer (Bio-Rad, USA), and sterilized ddH_2_O was applied as a control. The microbial growth curve was plotted with the absorbance value (OD_600_) as the ordinate and the incubation time as the abscissa. Vanillin standard curve equations were obtained from the correlation between absorbance value (OD_250_) and vanillin concentration. The vanillin concentration was calculated based on the standard curve. The degradation curve of vanillin was plotted with vanillin concentration as the ordinate and incubation time as the abscissa. While vanillic acid and cinnamic acid were initially included in the isolation experiment, only autotoxin-degrading bacteria exhibited growth in the presence of vanillin in MSM. This could be attributed to the possibility that the same concentration of vanillic acid and cinnamic acid led to a lower pH in MSM compared to vanillin. As a result, we proceeded with vanillin exclusively in our subsequent experiments.

**Transcriptome analysis**

2 mL of each strain solution was taken separately from MSM containing vanillin, which was added as the sole carbon source. The solution was centrifuged to collect microbes. Transferred bacterial sediment to a RNase-free centrifuge tube, added liquid nitrogen for quick freezing, and then stored it at -80°C for RNA extraction. MSM containing dextrose was used as a control. Each treatment contained 4 biological replicates. RNA was extracted using the TRIzol method (Invitrogen, USA) and treated with RNase-free DNase I (Takara, Japan). RNA degradation and contamination were monitored on 1% agarose gels. RNA concentration and purity were measured using a NanoDrop spectrophotometer. RNA quality and integrity were assessed using an Agilent 2100 Bioanalyzer. Using Ribo-off rRNA Depletion Kit (Bacteria) (Vazyme, China) to remove rRNA. Sequencing libraries were generated using the NEBNext® UltraTM RNA Library Prep Kit (New England Biolabs, USA). Library quality was assessed on an Agilent 2100 Bioanalyzer system. The library preparations were sequenced on the Illumina NovaSeq 6000 platform.

The adapter reads, N (uncertain base) reads and low-quality reads were removed from the raw data to obtain clean reads by using Trimmomatic. These clean reads were then mapped to the reference genome sequence by Bowtie 2. HTSeq [22] was used to count the read numbers mapped to each gene. Differential expression analysis of two groups was performed using the “DESeq2” package [23], and the *P*-value < 0.05 was adjusted by the Benjamini and Hochberg approach for controlling the false discovery rate (FDR) [24] to identify differentially expressed genes (DEGs). KEGG enrichment analysis of DEGs was implemented by the “clusterProfiler” package (*P*-value < 0.05) [25].

**Experiment 4:** **Verification of the alleviation of the replanting problem by autotoxin-degrading microbes**

**Phytotoxic bioassay**

Vanillin and the seeds of tobacco variety “K326” were used in a phytotoxic bioassay. Two isolated strains were separately incubated. The culture solution was subjected to centrifugation, followed by resuspension with sterile water. The resulting suspension was then adjusted to a cell density (OD_600_) of 1.0, corresponding to a colony count of 1 × 10^9^ cfu/mL. First, a solution of vanillin at a concentration of 0.5 mg/mL was introduced onto sterile Petri dishes with two layers of sterile filter paper. Second, strain solution, their mixture or sterilized distilled water (blank control) was added to filter paper, respectively. Third, tobacco seeds were placed separately in these Petri dishes, and incubated in darkness. Each treatment consisted of 4 biological replicates. Tobacco seed germination percentage, plant height, root length and fresh weight were determined after two (low vanillin concentration) or three (high vanillin concentration) weeks.

**Effects of phenolic acid and LTCC-differentially abundant microbes on tobacco seedling growth in soil**

Another pot experiment was conducted with the following five treatments: i) A: sterilized STCC soil with autotoxin, ii) A1: sterilized STCC soil with autotoxin and strain NLJ1, iii) A2: sterilized STCC soil with autotoxin and strain NLJ2, iv) AM: sterilized STCC soil with autotoxin and a mixture of strains NLJ1 and NLJ2, v) W: sterilized STCC soil with water. In total, there were 5 treatments × 8 replicates = 40 pots. Soil samples were taken from Niulanjiang Country (25º17’ N, 103º13’ E), Yunnan Province, China. The tobacco variety “K326” was planted continuously for 3 years (STCC) in fields. Soil collection method was the same as above-mentioned. STCC soil was autoclaved twice at 121°C for 30 min before use. The two isolated strains were individually incubated at 28°C and 180 rpm for 24 h. Following incubation, the culture solutions were subjected to centrifugation at 8000 rpm for 5 min, followed by resuspension with sterile water. Subsequently, the cell density was adjusted to an OD_600_ of 1.0, equivalent to a colony count of 1 × 10^9^ cfu/mL. The suspension of strains and their mixture were mixed separately with sterile soil in sterilized plastic bags (5%), and put in the dark for a week before the pot experiment.

The method of adding autotoxin (vanillin) to the soil, the management of the pot experiment, and the subsequent collection method of tobacco seedlings and soil samples were the same as in the aforementioned experiment 2. Metagenome sequencing and statistical analyses were also the same as in experiment 2. Sequence alignments were conducted using 16S rRNA gene sequences from two strains against gene sequences from metagenome sequencing.

**Statistical analyses**

Normalization of the sequencing data was performed using the total sum scaling [26]. For experiment 1: the difference in soil pH, phenolic acids, and relative abundances of microbial taxa (phyla) were compared between STCC and LTCC fields using a linear mixed model, with the region of the sample site used as a random factor. The analyses were conducted using the “nlme”, “multcomp” and “lsmeans” packages [27-29]. Means and standard errors were calculated for all treatments. Canonical analysis of principal coordinates (CAP) was visualized in the “vegan” and “ggplot2” [30] packages to evaluate variation in microbial community composition. The overall differences between different continuous cropping fields were compared with permutational multivariate analysis of variance (PERMANOVA) [31] in the “vegan” package. Spearman correlation analysis was used to identify correlations between phenolic acid contents and relative abundances of microbial taxa (phyla) in the “psych” package [32]. Heatmap figures were generated in the “pheatmap” package [33]. The different OTUs across samples were detected using the “edgeR” package [34] with a *P*-value < 0.05.

For experiment 2 and 4: plant biomass parameters and vanillin content were analyzed of different treatments by analysis of variance (ANOVA), and a mean comparison of treatments was performed based on Tukey’s honestly significant difference (HSD) test at the 0.05 probability level. Means and standard errors were calculated for all treatments. Principal coordinate analysis (PCoA) was based on Bray-Curtis dissimilarity matrices and visualized in the “vegan” and “ggplot2” packages to evaluate variation in microbial community and gene function composition. The overall differences of different treatments were compared with PERMANOVA in the “vegan” package. The different OTUs across samples were detected using the “edgeR” package with a *P*-value < 0.05. Spearman correlation analysis was used to identify correlations between phenolic acid contents and relative abundances of microbial taxa in the “psych” package. Heatmap figures were generated in the “pheatmap” package.

**References**

1. Dalton, Barry R., Sterling B. Weed, Udo Blum. 1987. “Plant phenolic acids in soils: a comparison of extraction procedures.” *Science Society of America Journal* 51: 1515-1521.

2. Li, Xiaogang, Changfeng Ding, Ke Hua, Taolin Zhang, Yanan Zhang, Ling Zhao, Yiru Yang, Jinguang Liu, Xingxiang Wang. 2014. “Soil sickness of peanuts is attributable to modifications in soil microbes induced by peanut root exudates rather than to direct allelopathy.” *Soil Biology and Biochemistry* 78: 149-159. <http://dx.doi.org/10.1016/j.soilbio.2014.07.019>

3. Lee, Charles K, Béatrice A Barbier, Eric M Bottos, Ian R McDonald, Stephen Craig Cary. 2012. “The inter-valley soil comparative survey: the ecology of dry valley edaphic microbial communities.” *The ISME Journal* 6: 1046-1057. <https://doi.org/10.1038/ismej.2011.170>

4. Chen, Lijun, Yuji Jiang, Chao Liang, Yu Luo, Qinsong Xu, Cheng Han, Qiguo Zhao, Bo Sun. 2019. “Competitive interaction with keystone taxa induced negative priming under biochar amendments.” *Microbiome* 7: 77. <https://doi.org/10.1186/s40168-019-0693-7>

5. Edgar, Robert C. 2013. “UPARSE: highly accurate OTU sequences from microbial amplicon reads.” *Nature Methods* 10: 996-998. <https://doi.org/10.1038/nmeth.2604>

6. Quast, Christian, Elmar Pruesse, Pelin Yilmaz, Jan Gerken, Timmy Schweer, Pablo Yarza, Jörg Peplies, Frank Oliver Glöckner. 2013. “The SILVA ribosomal RNA gene database project: improved data processing and web-based tools.” *Nucleic Acids Research* 41: D590-D596. <https://doi.org/10.1093/nar/gks1219>

7. Altschul, Stephen F., Warren Gish, Webb Miller, Eugene W. Myers, David J. Lipman. 1990. “Basic local alignment search tool.” *Journal of Molecular Biology* 215: 403-410. <https://doi.org/10.1016/S0022-2836(05)80360-2>

8. Kõljalg, Urmas, Karl-Henrik Larsson, Kessy Abarenkov, R. Henrik Nilsson, Ian J. Alexander, Ursula Eberhardt, Susanne Erland, et al. 2005. “UNITE: a database providing web-based methods for the molecular identification of ectomycorrhizal fungi.” *New Phytologist* 166: 1063-1068. <https://doi.org/10.1111/j.1469-8137.2005.01376.x>

9. Oksanen, Jari, F. Guillaume Blanchet, Roeland Kindt, Pierre Legendre, Peter R. Minchin, R. B. O’Hara, Gavin L. Simpson, et al. 2019. “vegan: Community ecology package.” R package version 2.5-6. <https://CRAN.R-project.org/package=vegan>

10. Blum, Udo, Steven R. Shafer. 1988. “Microbial populations and phenolic acids in soil.” *Soil Biology and Biochemistry* 20: 793-800.

11. Bolger, Anthony M., Marc Lohse, Bjoern Usadel. 2014. “Trimmomatic: a flexible trimmer for Illumina sequence data.” *Bioinformatics* 30: 2114-2120. <https://doi.org/10.1093/bioinformatics/btu170>

12. Li, Dinghua, Chi-Man Liu, Ruibang Luo, Kunihiko Sadakane, Tak-Wah Lam. 2015. “MEGAHIT: an ultra-fast single-node solution for large and complex metagenomics assembly via succinct *de Bruijn* graph.” *Bioinformatics* 31: 1674-1676. <https://doi.org/10.1093/bioinformatics/btv033>

13. Li, Weizhong, Adam Godzik. 2006. “Cd-hit: a fast program for clustering and comparing large sets of protein or nucleotide sequences.” *Bioinformatics* 22: 1658-1659. <https://doi.org/10.1093/bioinformatics/btl158>

14. Langmead, Ben, Steven L Salzberg. 2012. “Fast gapped-read alignment with Bowtie 2.” *Nature Methods* 9: 357-359. <https://doi.org/10.1038/nmeth.1923>

15. Li, Weizhong, Lukasz Jaroszewski, Adam Godzik. 2002. “Tolerating some redundancy significantly speeds up clustering of large protein databases.” *Bioinformatics* 18: 77-82. <https://doi.org/10.1093/bioinformatics/18.1.77>

16. Buchfink, Benjamin, Chao Xie, Daniel H Huson. 2015. “Fast and sensitive protein alignment using DIAMOND.” *Nature Methods* 12: 59-60. <https://doi.org/10.1038/nmeth.3176>

17. Ogata, Hiroyuki, Susumu Goto, Kazushige Sato, Wataru Fujibuchi, Hidemasa Bono, Minoru Kanehisa. 1999. “KEGG: Kyoto encyclopedia of genes and genomes.” *Nucleic Acids Research* 27: 29-34. <https://doi.org/10.1093/nar/27.1.29>

18. Johansen, Joachim, Damian R. Plichta, Jakob Nybo Nissen, Marie Louise Jespersen, Shiraz A. Shah, Ling Deng, Jakob Stokholm, et al. 2022. “Genome binning of viral entities from bulk metagenomics data.” *Nature Communications* 13: 965. <https://doi.org/10.1038/s41467-022-28581-5>

19. Parks, Donovan H., Michael Imelfort, Connor T. Skennerton, Philip Hugenholtz, Gene W. Tyson. 2015. “CheckM: assessing the quality of microbial genomes recovered from isolates, single cells, and metagenomes.” *Genome Research* 25: 1043-1055. <http://www.genome.org/cgi/doi/10.1101/gr.186072.114>

20. He, Hao, Wei Zhu, Iqra Noor, Junwei Liu, Guohuai Li. 2019. “*Pseudomonas putida* WH-B3 degrades benzoic acid and alleviates its autotoxicity to peach (*Prunus persica* L. batsch) seedlings grown in replanted soil.” *Scientia Horticulturae* 255: 183-192. <https://doi.org/10.1016/j.scienta.2019.05.020>

21. Kumar, Sudhir, Glen Stecher, Michael Li, Christina Knyaz, Koichiro Tamura. 2018. “MEGA X: molecular evolutionary genetics analysis across computing platforms.” *Molecular Biology and Evolution* 35: 1547-1549. <https://doi.org/10.1093/molbev/msy096>

22. Putri, Givanna H, Simon Anders, Paul Theodor Pyl, John E Pimanda, Fabio Zanini. 2022. “Analysing high-throughput sequencing data in Python with HTSeq 2.0.” *Bioinformatics* 38: 2943-2945. <https://doi.org/10.1093/bioinformatics/btac166>

23. Love, Michael I, Wolfgang Huber, Simon Anders. 2014. “Moderated estimation of fold change and dispersion for RNA-seq data with DESeq2.” *Genome Biology* 15: 550. <https://doi.org/10.1186/s13059-014-0550-8>

24. Benjamini, Yoav, Yosef Hochberg. 1995. “Controlling the false discovery rate: a practical and powerful approach to multiple testing.” *Journal of the Royal Statistical Society. Series B (Methodological)* 57: 289-300.

25. Yu, Guangchuang, Ligen Wang, Yanyan Han, and Qingyu He. 2012. “clusterProfiler: An R package for comparing biological themes among gene clusters.” *OMICS: a Journal of Integrative Biology* 16: 284-287. <https://doi.org/10.1089/omi.2011.0118>

26. Weiss, Sophie, Zhenjiang Zech Xu, Shyamal Peddada, Amnon Amir, Kyle Bittinger, Antonio Gonzalez, Catherine Lozupone, et al. 2017. “Normalization and microbial differential abundance strategies depend upon data characteristics.” *Microbiome* 5: 27. <https://doi.org/10.1186/s40168-017-0237-y>

27. Pinheiro, José, Douglas Bates, Saikat DebRoy, Deepayan Sarkar, the R Development Core Team. 2021. “nlme: Linear and nonlinear mixed effects models.” R package version 3.1-152. <https://CRAN.R-project.org/package=nlme>

28. Hothorn, Torsten, Frank Bretz, Peter Westfall. 2008. “Simultaneous inference in general parametric models.” *Biometrical Journal* 50: 346-363. <https://doi.org/10.1002/bimj.200810425>

29. Lenth, Russell V.. 2016. “Least-squares means: the R package lsmeans.” *Journal of Statistical Software* 69: 1-33. <https://doi.org/10.18637/jss.v069.i01>

30. Wickham, Hadley. 2016. “ggplot2: Elegant graphics for data analysis.” Springer-Verlag New York. <https://ggplot2.tidyverse.org>

31. Anderson, Marti J.. 2001. “A new method for non-parametric multivariate analysis of variance.” *Austral Ecology* 26: 32-46. <https://doi.org/10.1111/j.1442-9993.2001.01070.pp.x>

32. Revelle, William. 2020. “psych: Procedures for personality and psychological research.” <https://CRAN.R-project.org/package=psych>

33. Kolde, Raivo. 2019. “pheatmap: Pretty Heatmaps.” R package version 1.0.12. <https://CRAN.R-project.org/package=pheatmap>

34. Robinson, Mark D., Davis J. McCarthy, Gordon K. Smyth. 2010. “edgeR: A Bioconductor package for differential expression analysis of digital gene expression data.” *Bioinformatics* 26: 139-140. <https://doi.org/10.1093/bioinformatics/btp616>

**
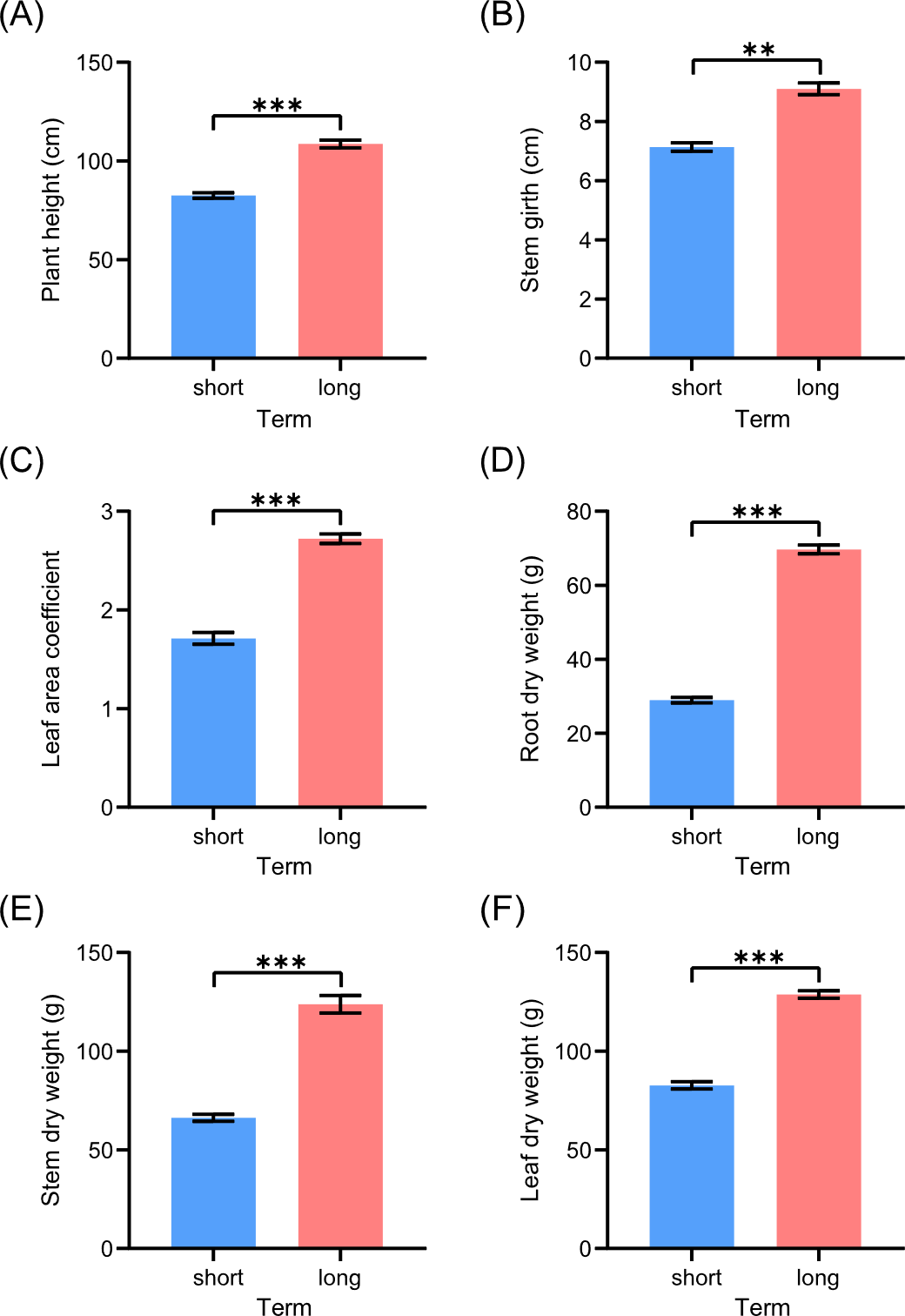
Figure S1 Tobacco plant growth in field soil in long-term continuous cropping (LTCC) and short-term continuous cropping (STCC) (n = 3).** (A) Plant height. (B) Stem girth. (C) Leaf area coefficient. (D) Root dry weight. (E) Stem dry weight. (F) Leaf dry weight. Means are compared with *t*-test. Error bars represent standard errors, ** indicates *p* < 0.01, *** indicates *p* < 0.001.

**
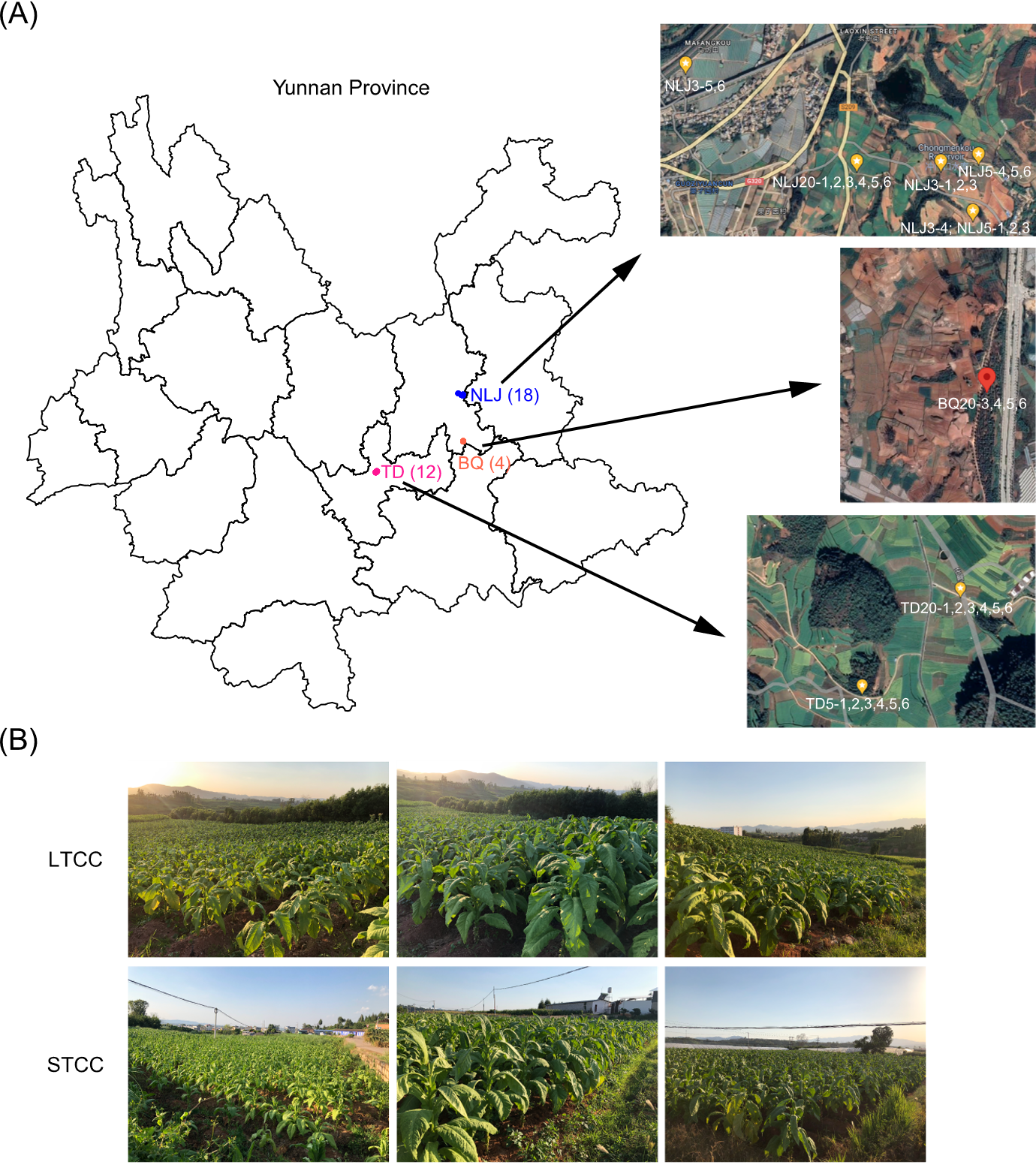
Figure S2 Location, distribution and tobacco growth of sampling sites in field soil.** (A) Location and distribution of sampling sites. Different colors represent different sampling locations. (B) Tobacco growth of sampling sites. NLJ, Niulanjiang; BQ, Banqiao; TD, Tadian. LTCC, long-term continuous cropping soil; STCC, short-term continuous cropping soil.

**
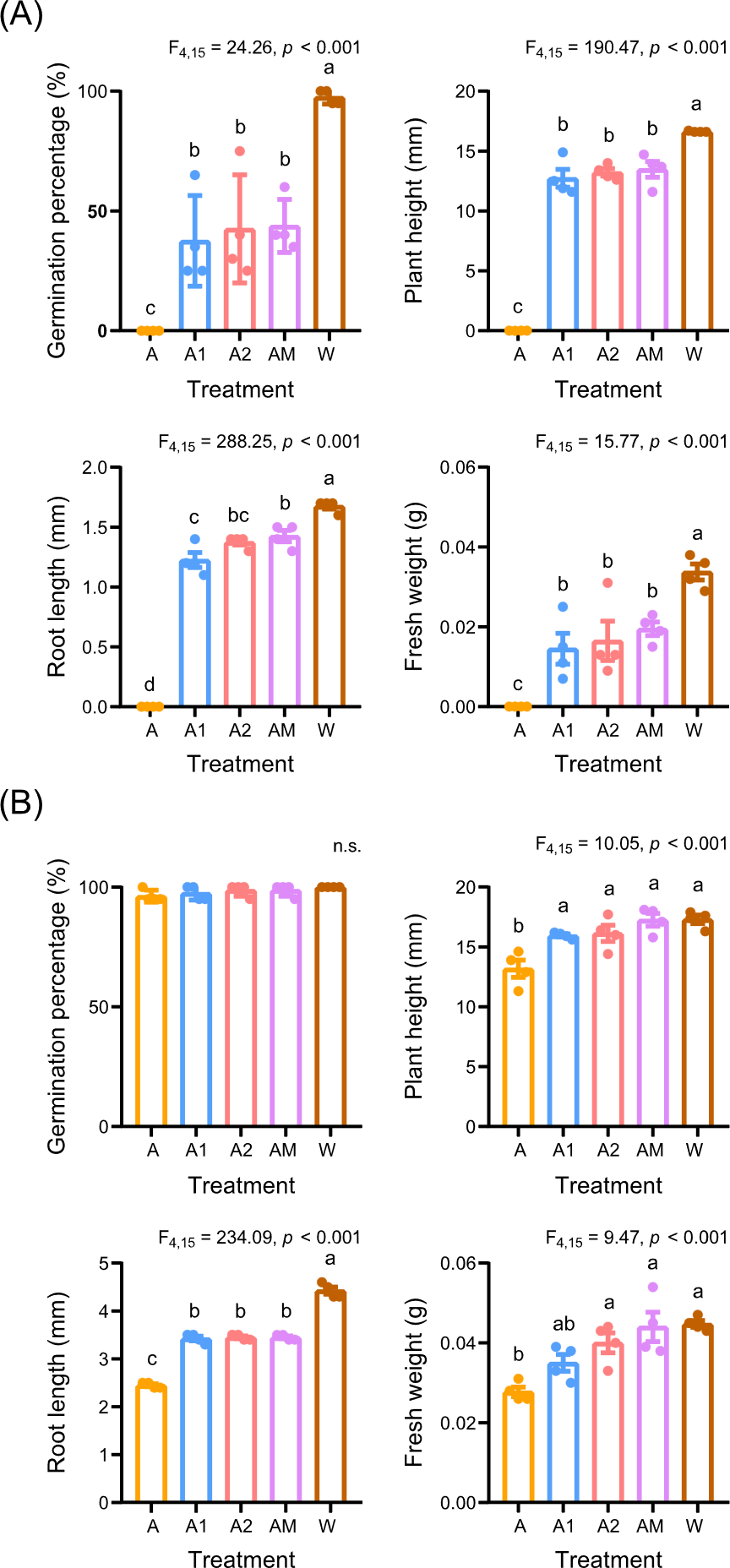
Figure S3 Effects of vanillin and degrading microbes on tobacco seeds** **germination and growth (n = 4).** (A) High vanillin concentration (1 mg/mL). (B) Low vanillin concentration (0.1 mg/mL). A, autotoxin; A1, autotoxin + NLJ1; A2, autotoxin + NLJ2; AM, autotoxin + NLJ1 + NLJ2; W, water.


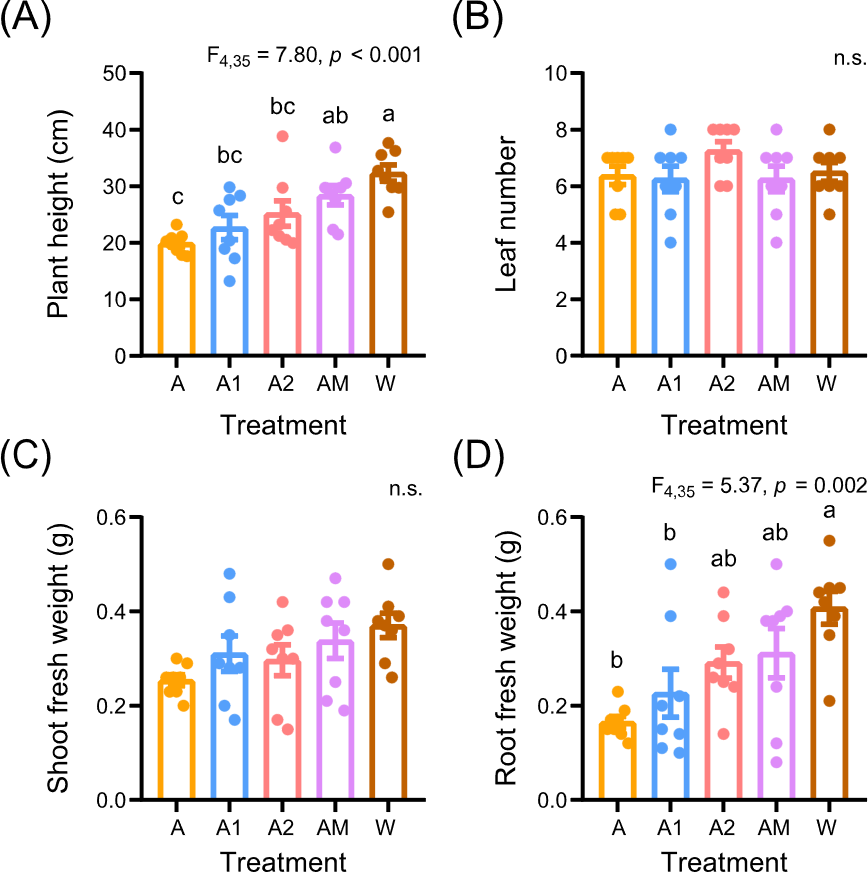
**Figure S4 Vanillin degradation and tobacco seedling growth in short-term continuous cropping (STCC) soil by inoculation of autotoxin-degrading bacteria NLJ1, NLJ2 and their combination (n = 8).** (A) Plant height. (B) Leaf number. (C) Shoot fresh weight. (D) Root fresh weight. A, autotoxin; A1, autotoxin + NLJ1; A2, autotoxin + NLJ2; AM, autotoxin + NLJ1 + NLJ2; W, water.


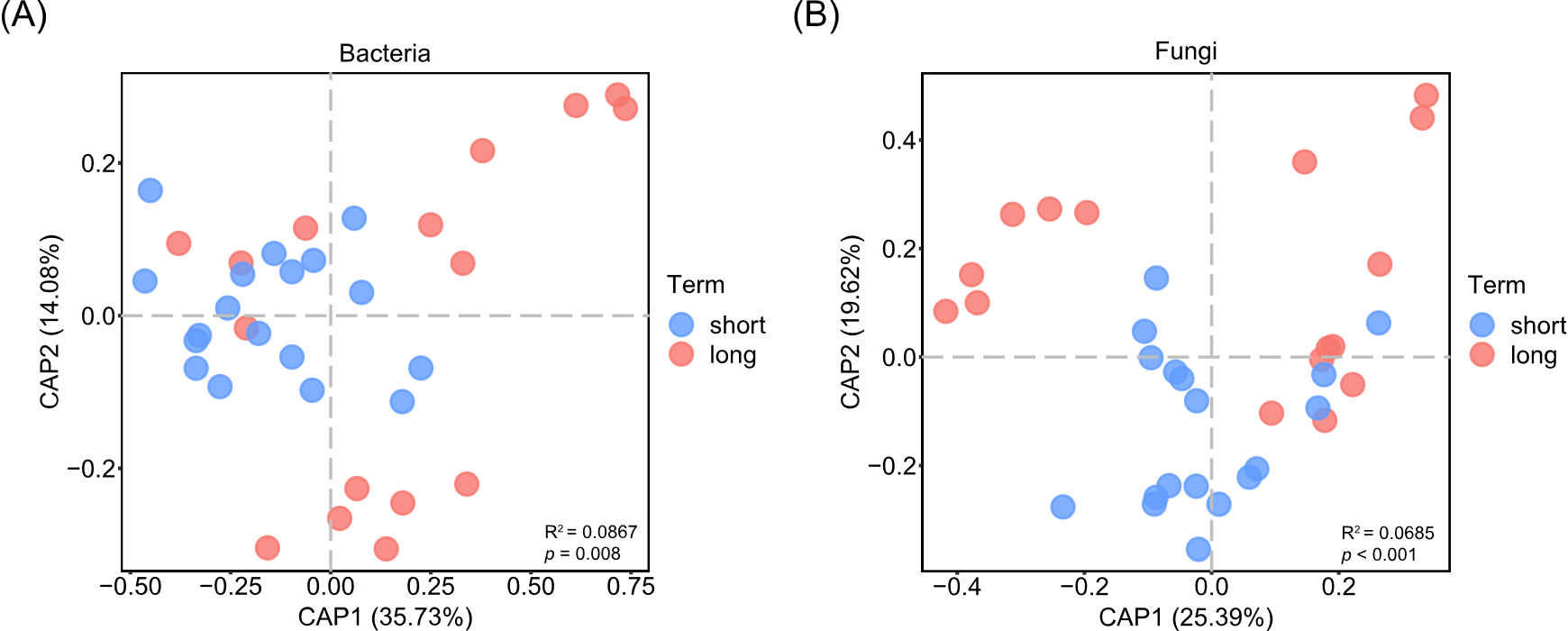
**Figure S5 Microbial community structure in tobacco field soil in long-term continuous cropping (LTCC, n = 16) and short-term continuous cropping (STCC, n = 18).** (A) Canonical analysis of principal coordinates (CAP) of bacterial and fungal (B) community structure.

**
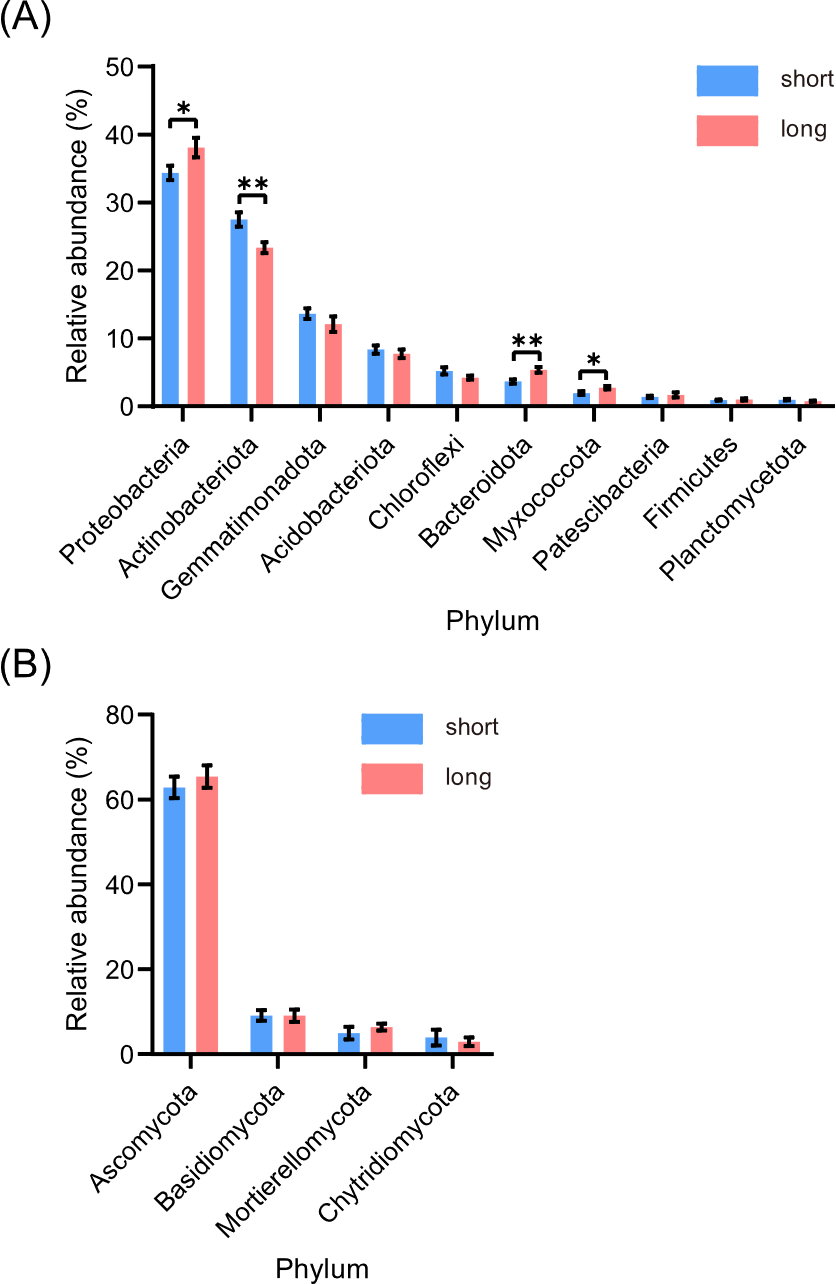
Figure S6 Microbial community composition in tobacco field soil in long-term continuous cropping (LTCC, n = 16) and short-term continuous cropping (STCC, n = 18).** (A) Relative abundances of bacterial and fungal (B) dominant phyla. Means are compared using a linear mixed model. Error bars represent standard errors, * indicates *p* < 0.05, ** indicates *p* < 0.01.


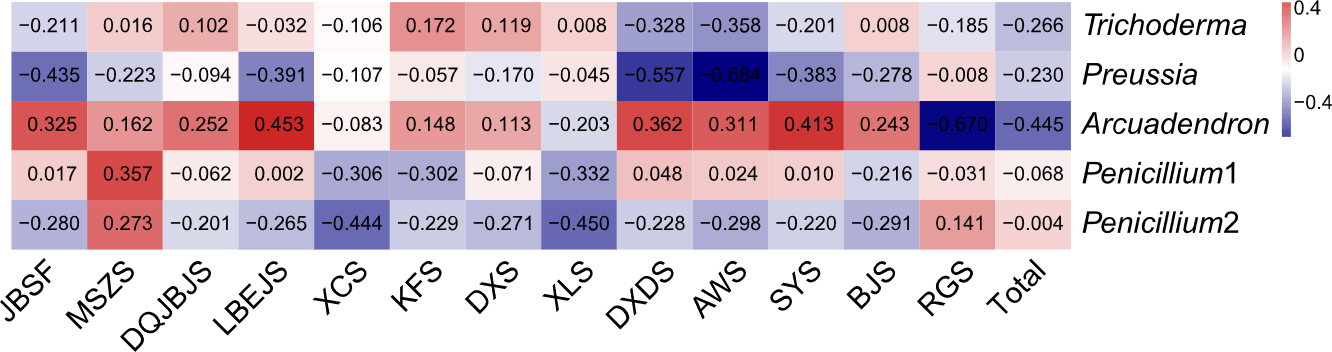
**Figure S7 Heatmap of Spearman’s correlation coefficients correlating fungal taxa enriched in long-term continuous cropping soil and phenolic acid.** JBSF, phloroglucinol; MSZS, gallic acid; DQJBJS, *p*-hydroxybenzoic acid; LBEJS, phthalic acid; XCS, vanillic acid; KFS, caffeic acid; DXS, syringic acid; XLS, vanillin; DXDS, *p*-coumaric acid; AWS, ferulic acid; SYS, salicylic acid; BJS, benzoic acid; RGS, cinnamic acid; Total, total phenolic acids.

**
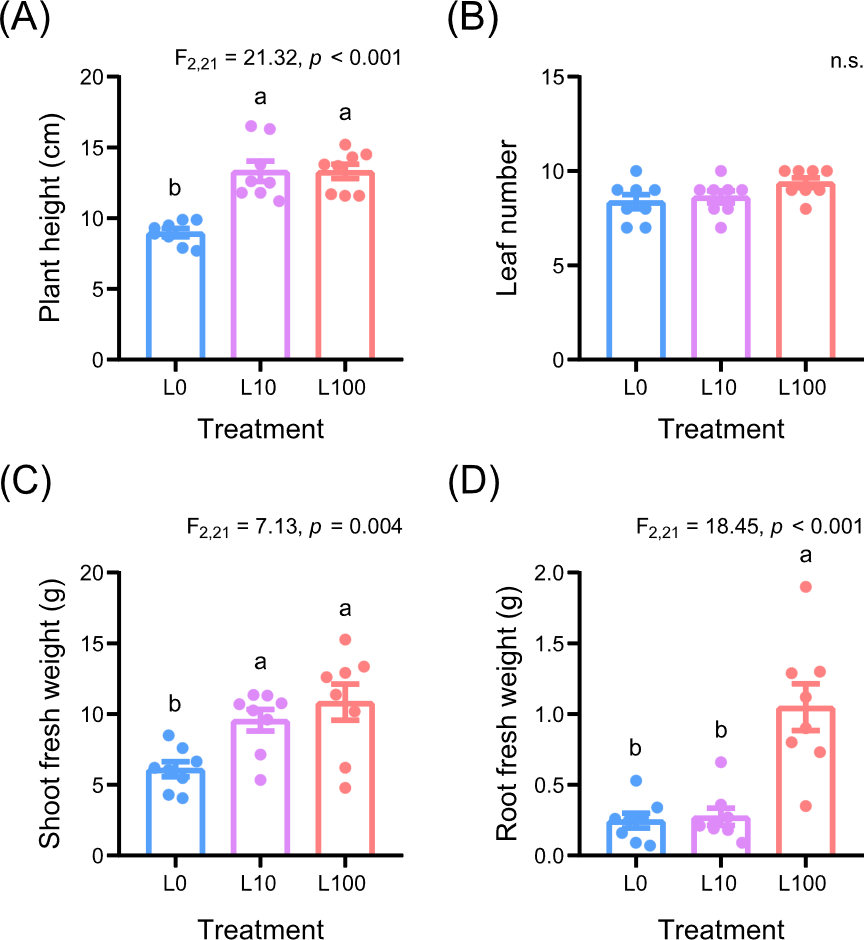
Figure S8 Effects of autotoxin on tobacco seedling growth in soil treated with autotoxin in different continuous cropping years (n = 8).** (A) Plant height. (B) Leaf number. (C) Shoot fresh weight. (D) Root fresh weight. L0, short-term continuous cropping soil (STCC); L10, short-term continuous cropping soil amended with 10% (w/w) of long-term continuous cropping soil; L100, long-term continuous cropping soil (LTCC).

**
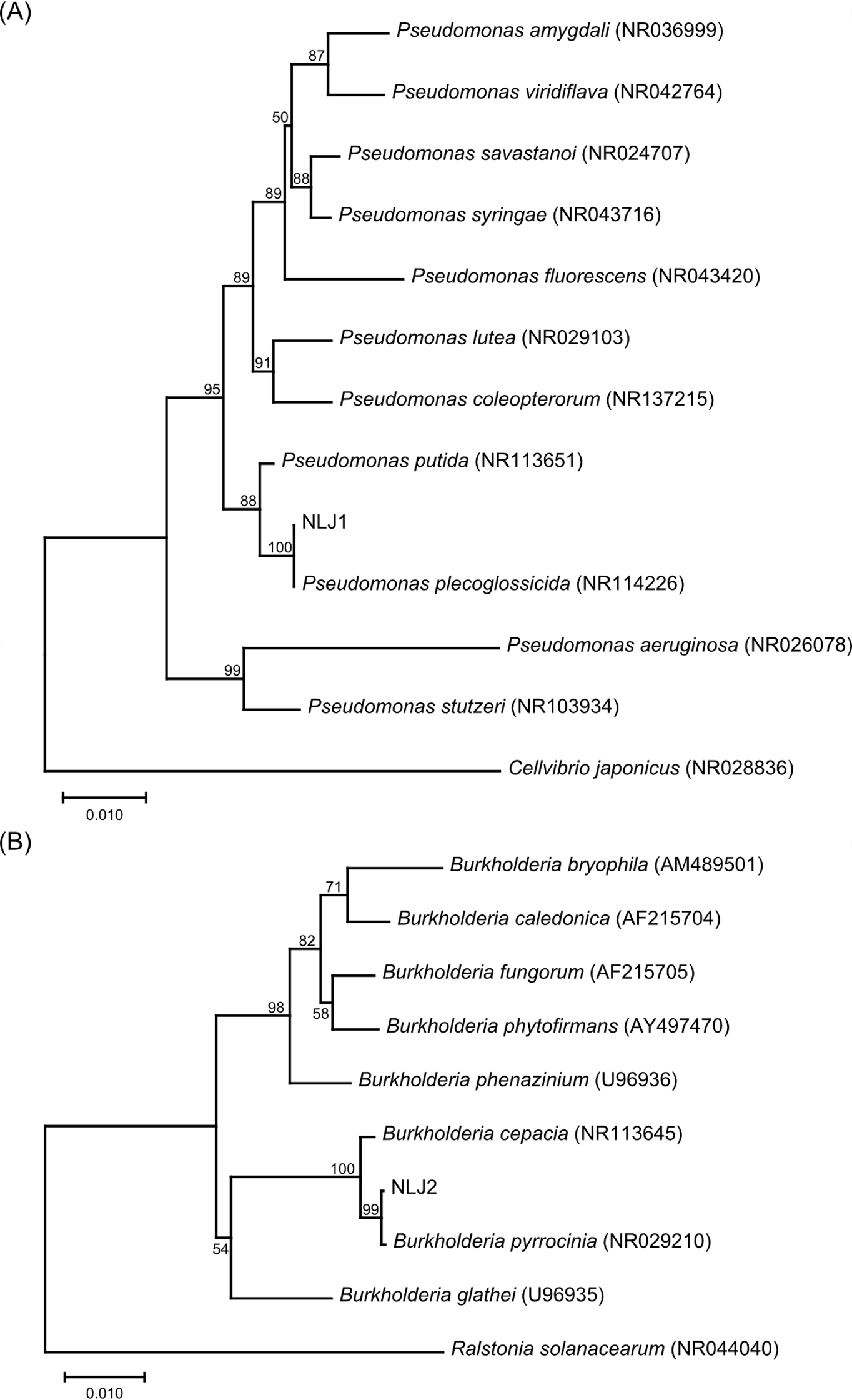
Figure S9 Phylogenetic tree of two strains and their closest sequences in GenBank based on 16S rRNA gene sequences.** Distances and clustering were performed using the neighbor-joining method. Sequence accession numbers of individual member strains are in parentheses. Numbers at branches indicate bootstrap values (percentages of 1000 replicates). Scale bar represents 0.01 substitutions per site. (A) *Cellvibrio japonicus* was used as the outgroup. (B) *Ralstonia solanacearum* was used as the outgroup.


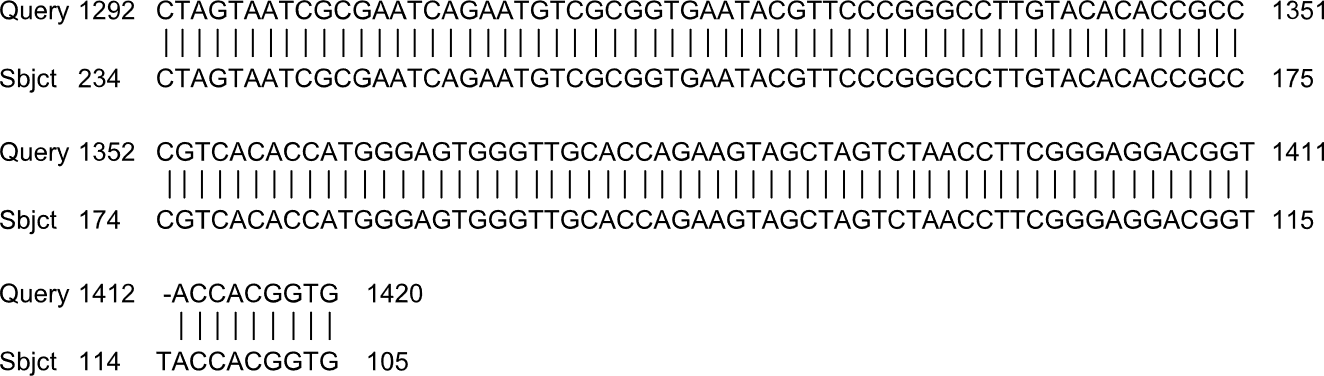
**Figure S10 Sequence alignment using 16S rRNA gene sequence of strain NLJ1 against gene sequence from metagenome sequencing in soil by inoculation of autotoxin-degrading bacteria.** Query sequence was 16S rRNA gene sequence of strain NLJ1. Subject sequence was gene sequence differentially abundant in tobacco soil treated with mixed strains.
